# Social cognition in children with epilepsy in mainstream education

**DOI:** 10.1111/dmcn.12613

**Published:** 2014-10-21

**Authors:** Adina R Lew, Charlie Lewis, Judith Lunn, Pamela Tomlin, Helen Basu, Julie Roach, Karl Rakshi, Timothy Martland

**Affiliations:** 1 Psychology Department, Lancaster UniversityLancaster, UK; 2 Paediatric Neurology Department, Royal Preston HospitalPreston, UK; 3 Paediatric Department, Royal Blackburn HospitalBlackburn, UK; 4 Paediatric Neurology Department, Royal Manchester Children's HospitalManchester, UK

## Abstract

**Aim:**

To establish whether deficits in social cognition are present in children with generalized or focal epilepsy in mainstream education, and whether any relation exists between social cognition, communication, and behaviour measures.

**Method:**

In a cross-sectional study, children with an epilepsy-only diagnoses in mainstream education (*n*=20 with generalized epilepsy; eight males, 12 females; mean age 11y 6mo, SD 2y 6mo; and *n*=27 with focal epilepsy; 12 males, 15 females; mean age 11y 8mo, SD 2y 2mo) and comparison participants (*n*=57; 28 males, 29 females; mean age 11y 2mo, SD 2y 4mo) were administered the Strange Stories task and the Mind in the Eyes task, as well as an IQ assessment. Parents completed the Children's Communication Checklist-2 and the Child Behavior Checklist (CBCL).

**Results:**

Both groups of children with epilepsy performed more poorly than control children on the Mental Stories component of the Strange Stories task, *F*(2,101)=3.2, *p*<0.001. Performance on Mental Stories was related to pragmatic communication, but only in the generalized epilepsy group (*r*=0.51, *p*=0.03, 95% CI=0.2–0.8). There were no differences between epilepsy groups or control participants in the Mind in the Eyes task, *F*(2,101)=0.4, *p*=0.4.

**Interpretation:**

Children with ‘epilepsy only’ are at risk of deficits in social cognition and may require appropriate support.

**What this paper adds:**

- Children with focal and generalized epilepsy have deficits in social cognition.
- Social cognition was associated with pragmatic communication in generalized epilepsy.

Recent research has indicated that cognitive and mental health comorbidities in epilepsy populations are ubiquitous, with several authors suggesting that underlying brain pathology causes both the epilepsy itself and the comorbidities, sometimes with somewhat independent natural histories.^1^,^2^ Although generally less severe than for children with symptomatic epilepsy, cognitive and mental health comorbidities still occur for children with ‘epilepsy-only’ diagnoses with well-controlled seizures attending mainstream education.^1^–^4^

While cognitive functions such as memory, attention, and executive skills have received extensive research attention in epilepsy populations,^1^,^2^ until recently social cognition has been relatively neglected. Broadly, social cognition concerns the ability to process and interpret social information, including the ability to infer intentions and beliefs in the service of predicting behaviour.^5^ Research with adults with refractory temporal lobe epilepsy^6^,^7^ and refractory frontal lobe epilepsy^8^ has demonstrated deficits in social cognition, with more basic emotion recognition also being compromised in refractory temporal lobe epilepsy.^9^–^12^

The aim of this study was to examine whether children with epilepsy, within a community sample attending mainstream education, show deficits in measures of social cognition, in particular reasoning about mental states (the Strange Stories task^13^), and inferring mental states from the eyes.^14^ To our knowledge, this is the first study to examine social cognition in children with epilepsy, using tasks that have been used in adult research. If difficulties with processing social information are identified in individual children, there is opportunity for early intervention to occur.^15^ The Strange Stories task was selected as it has a physical vignettes control condition for general comprehension and reasoning abilities built in to it, which examines understanding of physical causality, as opposed to the social vignettes condition where inferences about, for example, lies and faux pas are required. The Mind in the Eyes task^14^ draws on systems for interpreting emotions (thus linked to orbitofrontal and superior temporal sulcus functioning^16^), and so complements the narrative-based Strange Stories task. The children with epilepsy were divided into two groups, those with focal epilepsy and those with generalized epilepsy, as earlier research has indicated that children with focal epilepsies may have particular impairments in some aspects of communication,^17^ although with less difference between generalized and focal groups occurring on measures of pragmatic aspects of communication.^17^–^19^ We predicted that children with epilepsy would perform more poorly than age-matched control children on the social cognition tasks, but we did not have sufficient basis for predicting differences between the two epilepsy groups.

A second aim was to establish whether performance on the social cognition tasks was related to measures of pragmatic language^20^ and behaviour problems.^21^ A few studies have identified vulnerabilities in the pragmatic aspects of communication in community-based samples of children with epilepsy.^17^–^19^ In terms of relations between social cognition measures and behaviour problems, Goloubouff et al.^12^ reported a relation between lower performance on fear recognition and parental reports of behaviour problems, but this only occurred in children with right-sided, as opposed to left-sided, refractory temporal lobe epilepsy. Given this uncertain evidence base, we treat this aspect of the research as exploratory. Finally, we examined whether core clinical variables such as age at onset of epilepsy, seizure control, and number of antiepileptic drugs are related to measures of social cognition, communication, and behaviour. An earlier age at onset has been identified as a risk factor for lower IQ and behaviour problems in several, but not all, studies.^1^,^3^,^4^ Findings are also inconsistent with respect to the effects of seizure control on communication and behaviour.^3^,^4^,^17^ We thus expected a relation between lower IQ and an earlier age at onset, but made no directional predictions with respect to the other measures and clinical variables.

## Method

### Participants

**Figure 1:**
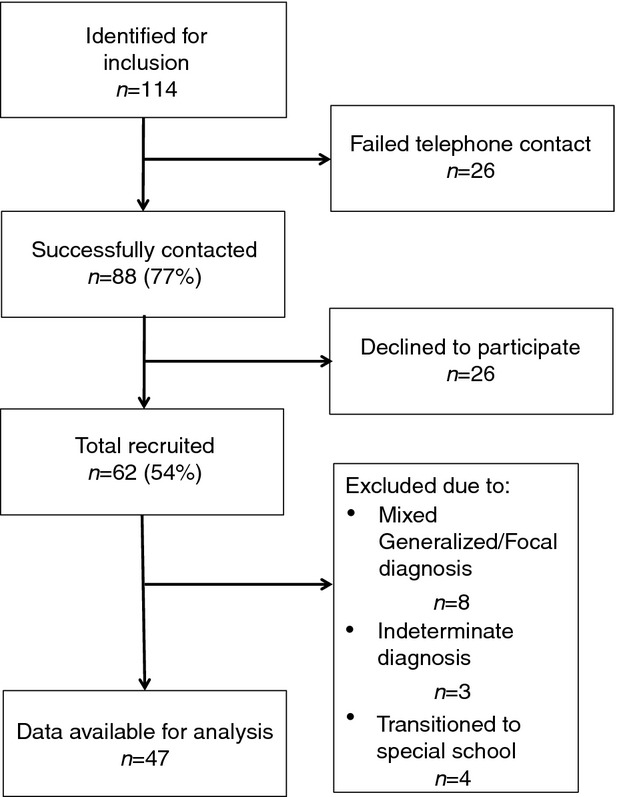
Participant recruitment and inclusion for the children with epilepsy.

The inclusion criteria for the children with epilepsy were children between 8 years and 16 years old in mainstream education with a diagnosis of epilepsy, excluding children with structural or metabolic aetiology. Children with epilepsy were identified from the paediatric neurology and general paediatric caseload of a hospital-based epilepsy specialist nurse (JR) in the north west of England. In November 2007, 114 children born between September 1991 and September 2002 were identified in the caseload, of which 85 fulfilled the study inclusion criteria. In order to further increase the sample, a paediatrician with a special interest in epilepsy from an adjacent area (KR), collated cases making clinic visits to community and hospital-based paediatricians between March and August 2009, which fitted the inclusion criteria. A further 29 children cases were referred for potential inclusion via this method, thus giving a total of 114 eligible cases. Figure1 provides further participant recruitment and inclusion data.

One consultant paediatric neurologist (PT) reviewed all the medical notes, with possible further discussion with two others (HB and TM). The epilepsy-related information is reported in TableI. Children with epilepsy were categorized into those with generalized epilepsy (*n*=20; eight males, 12 females; mean age 11y 6mo, SD 2y 6mo) and those with focal epilepsy (*n*=27; 12 males, 15 females; mean age 11y 8mo, SD 2y 2mo). Sixty-nine children (28 males, 29 females; mean age 11y 2mo, SD 2y 4mo) with no known neurological or neurodevelopmental condition were recruited from local mainstream schools concurrently with the testing of the children with epilepsy, in order to derive an age- and sex-matched control group of 57 children (birthday within 6 months of that of a child with epilepsy).

**Table I:** Participant demographic and clinical characteristics

Variables	Generalized epilepsy	Focal epilepsy	Comparison
	*n*=20	*n*=27	*n*=57
Sex Male/Female	8/12	12/15	28/29
Mean age, y:mo (SD)	11:6 (2:6)	11:8 (2:2)	11:2 (2:4)
Mean IQ (SD)	95.1 (16.8)	87.5 (14.1)	104.5 (12.8)
Ethnicity, white/Asian	18/2	24/3	52/5
Special educational support	5	13	0
SES^a^ Lower third rankings (%)	35	44	26
SES Middle third rankings (%)	25	26	25
SES Upper third rankings (%)	40	30	49
Age at onset, y:mo (SD)	7:2 (2:11)	6:10 (2:10)	
Duration, y:mo (SD)	3:4 (2:6)	3:4 (2:4)	
Seizure in last 6 months (%)	50	41	
Antiepileptic medication			
Monotherapy (%)	70	55	
Polytherapy (%)	11	20	
None (%)	19	25	
Epilepsy syndrome/type of epilepsy			
Childhood absence epilepsy	5		
Other	15		
Benign epilepsy with centrotemporal spikes		7	
Temporal		7	
Frontal		5	
Occipital		2	
Mixed focal		6	
Seizure types^b^			
Typical absence	11		
Tonic-clonic	12		
Myoclonic-atonic	1		
Atonic	1		
Consciousness unimpaired		2	
Consciousness impaired		15	
Evolving bilateral		11	

a Socioeconomic status (SES) as derived from the Indices of Deprivation rankings for postal code areas in England.

b Six children had two different seizure types.

A National Health Service Research Ethics Committee and a University Ethics Committee reviewed and approved the study. Written consent was gained from adult carers, and verbal or written consent was gained from child participants depending on their age.

### Measures

An estimated Full-scale IQ was derived from a short form of the Wechsler Intelligence Scale for Children IV-UK.^22^ The short form was comprised of Block Design, Similarities, Digit Span, and Coding. This short form has good internal reliability (0.932) as well as good validity (0.909) with Full-scale IQ.^23^

Two tasks were included as measures of social understanding, the revised version of the Strange Stories task^13^ and the child version of the Mind in the Eyes task.^14^ The Strange Stories task consists of 16 short passages each followed by a test question. Eight of the stories were constructed to measure the ability to infer mental states from scenarios involving lies, double bluff, white lies, persuasion, an appearance/reality distinction, and misunderstanding. The remaining eight stories were non-mental control stories that measured the understanding of physical events. A response was coded on a scale of 0 to 2 dependent upon whether the child gave a full, partial, or incorrect answer, with a maximum score of 16 for both Mental and Physical Stories.

The Mind in the Eyes task comprises 28 photographs of the eye region of male and female adults. The child was required to identify the correct option from four words or short phrases that are designed to assess the ability to correctly attribute both cognitive (e.g. ‘thinking about something’) or affective (e.g. ‘worried’) mental states. Scores range from 0 to 28.

The Child Communication Checklist-2^20^ (CCC-2) was used to assess language and communication. It is a standardized parental report questionnaire that is recommended as a screening tool for children who present with language impairments, pragmatic difficulties, or who may require further assessment for autistic spectrum disorder. The 70-item measure has 10 subscales, four that address aspects of structure, vocabulary, and discourse: Speech, Syntax, Semantics, and Coherence; four that explore the pragmatic aspects of language which include Inappropriate Initiation, Stereotyped Language, Use of Context, and Nonverbal Communication; and two scales that assess Social Relations and Interests included to screen for impairments characteristic of autistic spectrum disorder. The raw scores on the different scales are converted into standard scores with a mean of 10 and a standard deviation of 3, with high scores representing better reported language. The first eight scales can be added to produce a General Communication Composite score. A Social Interaction Deviance Composite score can also be derived from the discrepancy between measures of the structural and pragmatic aspects of language, combined with the two scales concerned with social interaction and interests. The Social Interaction Deviance Composite score is used to categorize children with profiles suggestive of specific language impairment, pragmatic language impairment, or autism spectrum disorders. Cronbach's alphas range between 0.66 and 0.8 for internal consistency of the 10 subscales,^20^ and the CCC-2 has high levels of validity in terms of discriminating children with and without a diagnosed language impairment^24^.

The Child Behavior Checklist (CBCL), 6 to 18 years,^21^ was used to assess behaviour problems. It is a standardized parental report questionnaire that includes 113 items which assess a wide range of behavioural difficulties. It provides three broad indices (termed broadband) of Internalizing, Externalizing, and Total Problems. The raw scores are converted to standardized T scores with a mean of 50 and a standard deviation of 10, with higher scores representing greater behaviour difficulties. The CBCL has high internal consistency (Cronbach's alpha for Total Problems=0.97), as well as high validity in terms of identifying clinical cases of conduct disorder.^21^

### Procedure

Children were either visited at school or at home dependent on parental preference. The assessments in the session with children were administered in the same order: Strange Stories, IQ, and Mind in the Eyes. Parents filled in questionnaires during home visits or returned them to the school if testing had been carried out at school.

### Statistical analyses

Group differences on the social cognition measures (Mental Stories, control Physical Stories, Emotion in the Eyes) were analysed using analysis of variance (ANOVA). All significance tests were two-tailed. These analyses are of interest in that the majority of children with epilepsy had no special support at school, the default assumption being that they are similar to peers both socially and academically. However, in terms of understanding whether any differences between epilepsy groups and controls on the social cognition tasks could be accounted for by differences in IQ, analyses taking into account IQ differences between groups are required. Analysis of covariance (ANCOVA) was not appropriate given group differences in IQ^25^ (see TableI). Thus, a general linear modelling forward-fitting approach was adopted to examine the effects of age, IQ, group (generalized epilepsy, focal epilepsy, and control participants), and their interaction on any social cognition measures showing group differences, with the Bayesian Information Criterion Index being used to ascertain the best-fitting model.

A 3(group) × 10(CCC-2 subscale) multivariate analysis of variance (MANOVA) with Bonferroni adjusted post hoc tests was used to compare performance in the different groups on the 10 subscales of the CCC-2. Univariate ANOVAs were used to compare groups on the summary scales of the CCC-2 and the CBCL. Again, a general linear modelling approach was utilized to account for IQ when examining group differences on the standardized language and behaviour measures, with just the summary scales being modelled in these analyses.

It should be noted that children were selected if they were attending mainstream education in the present study, with no IQ cut-off specified a priori for study inclusion. Five children were found to have IQs below 70 (three children with focal epilepsy, and two with generalized epilepsy), the cut-off used in earlier studies of epilepsy-only paediatric populations.^3^ We ran all analyses excluding these participants, but obtained the same pattern of results. We thus only report analyses including all participants. There were also no significant differences between home- and school-tested participants.

## Results

### Preliminary analyses

There was no significant difference between participants and non-participants in terms of socio-economic status (divided into upper, middle, and lower thirds) using the Indices of Deprivation rankings for postal code areas in England (χ^2^(2)=4.03, *p*=0.13). In terms of the coding of responses in the Strange Stories task, a second coder, blind to group assignment and study hypotheses, examined 50% of responses, with interobserver agreement found to be high (κ=0.81).

### Social cognition

**Figure 2:**
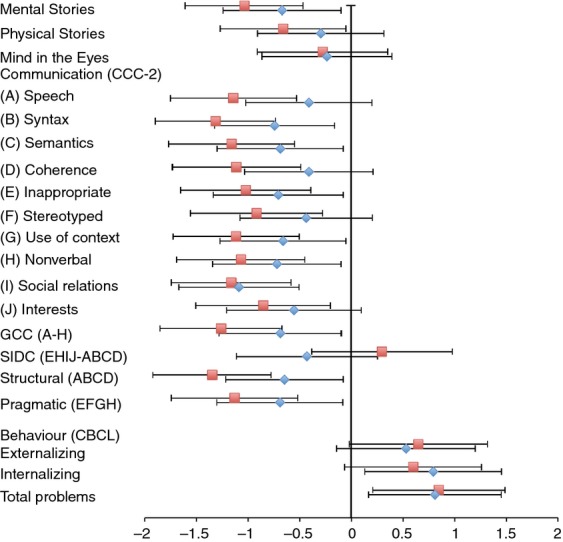
Mean differences (and 95% CIs) for generalized epilepsy versus control groups (blue diamonds), and focal epilepsy versus control groups (red squares), in social cognition, communication, and behaviour measures. All differences are expressed as standard scores. CBCL, Child Behavior Checklist; CCC-2, Child Communication Checklist-2.

TableII displays the means (SDs) for the generalized epilepsy, focal epilepsy, and control groups for the Mental Stories, Physical Stories, and the Mind in the Eyes tests, with mean differences (95% confidence interval [CI]) between epilepsy groups and control participants displayed in Figure2. Both the generalized epilepsy and focal epilepsy groups performed significantly worse than the control group on the Mental Stories test, whereas only the focal epilepsy group performed worse than the control group on the Physical Stories test. When age and IQ were included as covariates in a general linear model (TableIII), the best-fitting model for the Physical Stories only contained age and IQ as main effects, suggesting the worse performance of the focal epilepsy group, relative to the control group, could be accounted for by their lower IQ. In contrast, for the Mental Stories test, the best-fitting model contained the main effect of group, as well as the main effects of age and IQ, suggesting that group differences remained even accounting for age and IQ. Both epilepsy groups were significantly different from the control group, but were not significantly different from each other. There were no group differences on the Mind in the Eyes test.

**Table II:** Group means (SDs) for the measures of social cognition, communication, and behaviour

	Generalized	Focal	Comparison
Mental Stories	10.9 (3.4)	9.7 (3.5)	13.1 (2.4)
Physical Stories	9.9 (3.7)	8.6 (4.1)	11.0 (3.3)
Mind in the Eyes	17.2 (3.9)	17.0 (3.6)	17.9 (3.2)
Child Communication Checklist-2			
(A) Speech	8.4 (3.5)	5.9 (3.1)	9.8 (2.5)
(B) Syntax	7.9 (3.8)	5.9 (3.3)	10.5 (2.0)
(C) Semantics	7.7 (3.8)	5.9 (3.4)	10.4 (3.2)
(D) Coherence	8.6 (3.8)	6.0 (3.3)	10.1 (2.9)
(E) Inappropriate	8.0 (3.6)	6.8 (3.3)	10.5 (3.3)
(F) Stereotyped	8.3 (3.7)	6.8 (2.9)	9.8 (3.1)
(G) Use of context	7.3 (4.0)	5.4 (3.8)	10.1 (3.0)
(H) Nonverbal	7.5 (3.2)	6.2 (3.6)	9.8 (3.0)
(I) Social Relations	6.6 (3.4)	6.3 (4.2)	10.8 (2.7)
(J) Interests	8.3 (3.2)	7.2 (3.5)	10.2 (3.3)
GCC (A–H)	63.6 (25.1)	48.8 (23.3)	81.2 (17.9)
SIDC (EHIJ-ABCD)	–2.4 (8.6)	3.1 (6.9)	.8 (6.7)
Structural (ABCD)	32.6 (12.8)	23.6 (11.8)	40.8 (7.5)
Pragmatic (EFGH)	31.0 (13.4)	25.0 (12.4)	40.4 (11.0)
Child Behavior Checklist (CBCL)			
Externalizing	53.8 (13.2)	55.2 (12.3)	47.7 (8.7)
Internalizing	58.1 (12.3)	55.6 (13.4)	47.8 (11.3)
Total problems	56.8 (12.7)	57.3 (13.0)	46.3 (10.5)

*n*=33 in the control group for the CCC-2 and *n*=32 for the CBCL, due to non-returns. GCC, General Communication Composite; SIDC, Social Interaction Deviance Composite.

**Table III:** Parameter estimates for best-fit general linear models of Mental Stories, Physical Stories, structural language, pragmatic language and behaviour, with group, IQ, and age (for non-standardized measures) as predictors

Measure	Parameter	Estimate (β)	SE	95% CI	*p*	*R*^2^
Mental Stories^a^	Age	0.04	0.009	0.02–0.06	<0.001	0.46
	IQ	0.08	0.02	0.04–0.11	<0.001	
	Generalized epilepsy	–1.6	0.64	–2.8 to –0.3	0.002	
	Focal epilepsy	–2.3	0.63	–3.5 to –1.0	<0.001	
Physical Stories	Age	0.07	0.01	0.05–0.09	<0.001	0.43
	IQ	0.09	0.02	0.05–0.12	<0.001	
Structural language	IQ	0.2	0.08	0.06–0.36	0.006	0.40
	Generalized epilepsy	–6.5	2.9	–12.1 to –0.83	0.03	
	Focal epilepsy	–13.8	2.8	–19.4 to –8.3	<0.001	
Pragmatic language	Generalized epilepsy	–9.5	3.4	–16.0 to –2.9	0.005	0.24
	Focal epilepsy	–15.5	3.1	–21.5 to –9.4	<0.001	
Total Problems	Generalized epilepsy	10.5	3.3	3.9–17.0	0.002	0.17
	Focal epilepsy	11.0	3.1	5.0–17.0	<0.001	

The control group is used as the reference category in all analyses. For all models reported, the residuals did not depart from normality, and there were no dependencies between residuals and factors.

a A model which included group as a main effect, and an age and IQ interaction term only, was also a good fit, but given the focus on whether a group effect remains after controlling for age and IQ, the more interpretable main-effects model is reported. SE, standard error; CI, confidence interval.

### The Child Communication Checklist

TableII displays the mean (SD) scores for the 10 subscales of the CCC-2, the General Communication Composite score, the Social Interaction Deviance score, and the Pragmatics and Structural summary scales for the three groups, with mean differences (95% CI) between epilepsy groups and controls displayed in Figure2. TableIII shows the best-fitting general linear models including IQ as a covariate. Overall, both the generalized epilepsy and focal epilepsy groups had lower scores than the control children, with the children with focal epilepsy being particularly impaired in the structural language measures, as has been noted in earlier research.^17^,^18^ When IQ was included in the analyses (TableIII), the best-fitting model for the Structural summary scale included IQ as a main effect, together with group as a main effect whereby children with focal epilepsy were significantly worse than children with generalized epilepsy, who in turn were worse than control children. For pragmatic language, the best-fitting model included group only, whereby both the generalized epilepsy and focal epilepsy groups were worse than control children.

### The Child Behavior Checklist

TableII displays the mean scores and Figure2 shows mean differences (95% CI) between the two epilepsy groups and the control group, for the broadband psychopathology scales and the Total Problems scores. As with other research with similar samples of children with epilepsy,^3^ both groups of children with epilepsy showed higher levels of behaviour problems relative to the control group, and including IQ did not add significant explanatory power (TableIII).

### Relations between social cognition tasks and communication and behaviour

Partial correlations, controlling for age and IQ, were conducted between the Strange Stories measures, the Mind in the Eyes measure, and the four composite measures of the CCC-2, as well as the Total Problems measure of the CBCL. Correlation coefficients are reported in Table SI (see online supporting information). The Mind in the Eyes task did not correlate with any of the parental report measures. The Mental Stories of the Strange Stories task correlated with the General Communication Composite (*r*=0.49, *p*=0.04, 95% CI=0.1–0.8) and pragmatic language measures (*r*=0.51, *p*=0.03, 95% CI=0.2–0.8) but this was only the case in the generalized epilepsy group. There was no significant correlation with behaviour problems. Performance on the Physical Stories did not correlate with the communication or behaviour measures.

### Effects of clinical variables on social cognition, communication, and behaviour

The relation between age at onset and measures of social cognition, communication and behaviour problems was examined using partial correlations controlling for age and IQ, as a later age at onset was significantly positively related to both of these variables (age: *r*=0.6, *p*<0.001, 95% CI 0.4–0.8; IQ, *r*=0.37, *p*=0.01, 95% CI 0.01–0.6). The two epilepsy groups were pooled to gain power. The only significant correlation showed that later onset was related to more Total Problems (*r*=0.33, *p*=0.03, 95% CI 0.07–0.6). One-way ANOVAs with the three antiepileptic drug groups (polytherapy, monotherapy, and none) as levels of the factor, against social cognition, communication, and Total Problems measures yielded no significant effects. A comparison of those children who had experienced a seizure in the last 6 months versus those who had not experienced a seizure also yielded no significant differences. Finally, all group differences in Strange Stories performance, as well as summary language and behaviour measures, remained significant if only the children not in receipt of educational support were included in analyses, suggesting a level of unmet need in the sample.

## Discussion

We hypothesised that children with epilepsy-only diagnoses within mainstream education may have difficulties relative to their non-affected peers in tasks of social understanding. This was the case for the Strange Stories task, but not the Mind in the Eyes task. The lack of significant differences in performance on the Mind in the Eyes task between epilepsy and control groups appears to be in contrast to the findings with adults with refractory frontal lobe epilepsy.^8^ According to Sabbagh,^16^ this task is particularly dependent on orbitofrontal cortex functioning, and so it may be that epilepsy syndromes where this region is compromised show greater deficits relative to other types of epilepsy syndromes, a hypothesis that would require testing with larger samples of children and adults with different types of focal epilepsies.

As expected from earlier research, the children with epilepsy had poorer structural and pragmatic communication skills compared with their non-affected peers, as well as higher rates of behaviour problems.^3^,^4^,^17^–^19^ There was a relation between scores on Mental Stories and communication problems, but this pertained to the children with generalized epilepsy only. It is possible that the structural language difficulties in the children with focal epilepsy made it harder for parents to distinguish communication failures due to structural language problems as opposed to pragmatic problems, thus weakening the linkage between social cognition abilities and good pragmatic communication (see discussion of this issue in Broeders et al.^19^). It is also possible that the relation between social cognition and communication found in the generalized epilepsy group was not a reliable effect given the relatively small sample size in the present study, making further research desirable.

In terms of the relation between social cognition and behaviour, no significant correlation was found once IQ and age were controlled for. Taken together with the findings of Golouboff et al.,^12^ where only one of the temporal lobe epilepsy subgroups showed a relation between fear recognition and behaviour problems, it is clear that further replications across diverse epilepsy syndromes are required to establish whether any robust relations are present.

Finally, in terms of epilepsy variables, an earlier age at onset was related to lower IQ scores, as has been found in earlier research.^1^ Surprisingly however, a later age at onset was related to more behaviour problems, controlling for age and IQ. It is possible that some children with later onset epilepsy were still going through an adjustment period in terms of their epilepsy, in that Austin et al.^3^ found that behaviour generally improved 1 to 2 years after diagnosis, but low power prevented us from examining this possibility quantitatively. There was no additional deficit in social cognition associated with an earlier age at onset, over and above those related to lower IQ and age. Different types of epilepsy may vary in terms of whether age at onset is a risk factor in particular cognitive functions,^1^,^2^ but our results suggest that educators do not need to treat early age at onset as an additional risk factor for social cognition deficits.

Limitations of the present study include a relatively small sample size, such that there was insufficient power to examine whether subgroups of children with different types of epilepsies, within focal and generalized groupings, showed different social cognition profiles. Caution is also required because of the large number of measures examined, relative to the sample sizes of the clinical groups. Despite these limitations, educators and clinicians working with children with epilepsy should consider these children to be at risk of difficulties with understanding the thoughts and motivations of others. A specialist epilepsy nurse is well placed to inform and support schools, as well as the children and their families, in recognizing the difficulties that children with epilepsy may have in social understanding.

## Abbreviations

CCC-2: Child Communication Checklist-2
CBCL: Child Behavior Checklist

## Supporting Information

The following additional material may be found online:

Table SI Partial correlations (controlling for age and IQ) for social cognition, language and behaviour measures.
