# Supplementary material for: Social cognition in children with epilepsy in mainstream education
Source: Dev Med Child Neurol. 2014 Oct 21;57(1):53–9. doi: 10.1111/dmcn.12613 (PMC4328452; doi:10.1111/dmcn.12613)
Supplement: Table SI — Partial correlations (controlling for age and IQ) for social cognition, language and behaviour measures. [file dmcn0057-0053-sd1.docx]

**Table SI:** Partial correlations (controlling for Age and IQ) for social cognition, language and behaviour measures

|  | Parental Report Measures of Communication and Behaviour | | | | |
| --- | --- | --- | --- | --- | --- |
| Social Cognition Tasks | GCC  (CCC-2) | SIDC  (CCC-2) | Structural  (CCC-2) | Pragmatic  (CCC-2) | Total Problems (CBCL) |
| Control Group | | | | | |
| Mental Stories | -.13 | -.04 | -.08 | -.16 | .004 |
| Physical Stories | .07 | -.13 | .14 | .02 | -.04 |
| Eyes Task | .08 | -.11 | .08 | .08 | .27 |
| Generalised Epilepsy Group | | | | | |
| Mental Stories | .49, *p*=.04 | -.08 | .42 | .51, *p*=.03 | -.42 |
| Physical Stories | .22 | -.02 | .16 | .26 | .07 |
| Eyes Task | .25 | -.008 | .30 | .19 | -.15 |
| Focal Epilepsy Group | | | | | |
| Mental Stories | .15 | -.26 | .24 | .03 | .17 |
| Physical Stories | .26 | .03 | .23 | .24 | -.12 |
| Eyes Task | .17 | -.14 | .23 | .13 | -.004 |
